# Cannabinerol (CBNR) Influences Synaptic Genes Associated with Cytoskeleton and Ion Channels in NSC-34 Cell Line: A Transcriptomic Study

**DOI:** 10.3390/biomedicines12010189

**Published:** 2024-01-15

**Authors:** Osvaldo Artimagnella, Emanuela Mazzon, Stefano Salamone, Federica Pollastro, Agnese Gugliandolo, Luigi Chiricosta

**Affiliations:** 1 IRCCS Centro Neurolesi “Bonino-Pulejo”, Via Provinciale Palermo, Contrada Casazza, 98124 Messina, Italyemanuela.mazzon@irccsme.it (E.M.);; 2 Department of Pharmaceutical Sciences, University of Eastern Piedmont, Largo Donegani 2, 28100 Novara, Italy; salamone.ste@gmail.com (S.S.); federica.pollastro@uniupo.it (F.P.)

**Keywords:** cannabinerol, synapse, phytocannabinoids, cytoskeleton, ion channels, transcriptomic analysis

## Abstract

Cannabinoids are receiving great attention as a novel approach in the treatment of cognitive and motor disabilities, which characterize neurological disorders. To date, over 100 phytocannabinoids have been extracted from *Cannabis sativa*, and some of them have shown neuroprotective properties and the capacity to influence synaptic transmission. In this study, we investigated the effects of a less-known phytocannabinoid, cannabinerol (CBNR), on neuronal physiology. Using the NSC-34 motor-neuron-like cell line and next-generation sequencing analysis, we discovered that CBNR influences synaptic genes associated with synapse organization and specialization, including genes related to the cytoskeleton and ion channels. Specifically, the calcium, sodium, and potassium channel subunits (*Cacna1b*, *Cacna1c*, *Cacnb1*, *Grin1*, *Scn8a*, *Kcnc1*, *Kcnj9*) were upregulated, along with genes related to NMDAR (*Agap3*, *Syngap1*) and calcium (*Cabp1*, *Camkv*) signaling. Moreover, cytoskeletal and cytoskeleton-associated genes (*Actn2*, *Ina*, *Trio*, *Marcks*, *Bsn*, *Rtn4*, *Dgkz*, *Htt*) were also regulated by CBNR. These findings highlight the important role played by CBNR in the regulation of synaptogenesis and synaptic transmission, suggesting the need for further studies to evaluate the neuroprotective role of CBNR in the treatment of synaptic dysfunctions that characterize motor disabilities in many neurological disorders.

## 1. Introduction

Neurological disorders, including neurodegenerative diseases and traumatic brain injuries, represent a growing global health challenge, affecting up to one billion people worldwide and leading to significant impairments in cognitive and motor functions [1,2]. These diseases are characterized by the progressive and unstoppable loss of neurons and synapses, leading to functional decline. In this context, several studies support the effectiveness of synapse-targeting therapies that may ameliorate synaptic function in association with a delay in degeneration [3]. Currently, various treatments are available to counteract different neurological disorders, but there is no cure. In this context, cannabinoids have been shown to provide a novel approach to rehabilitation and treatment [4]. Δ^9^-Tetrahydrocannabinol (Δ^9^-THC), extracted from the *Cannabis sativa*, is one of the most famous and abundant phytocannabinoids, which is a term used to refer to plant-derived cannabinoids. The discovery of Δ^9^-THC in 1971 led to the identification of the endocannabinoid system (ES), which includes CB1 and CB2 receptors and is fundamental in the neuromodulation of the central nervous system. However, the administration of Δ^9^-THC also results in psychotropic effects that have considerably restricted its clinical applicability [5]. In addition to CB1 and CB2 receptors, cannabinoids can also bind other receptors, namely peroxisome-proliferator-activated receptors (PPARs), the GTP-binding-protein-coupled receptor GPR55, and transient receptor potential vanilloid type 1 (TRPV1) cation channels (reviewed in [6]).

The cannabis plant is the source of over 100 phytocannabinoids; some of them show no psychotropic consequences and, for this reason, are of potential interest in the treatment of neurological disorders. Previously, research in the phytocannabinoid area was mainly focused on the so-called “Big Four”, which is a term referring to Δ^9^-THC, cannabidiol (CBD), cannabigerol (CBG), and cannabichromene (CBC). CBD and CBG have already shown neuroprotective qualities in rodents [4]. Indeed, in vivo experiments have demonstrated the efficacy of CBD in restoring neurobehavioral function, decreasing neurological deficits, and improving neuroplasticity and working memory [7,8]. Similarly, CBG improved motor activities, prevented neuronal loss, and decreased inflammatory mediators in in vivo experimental models [9]. Moreover, in a previous study, we reported that CBG and CBD influence the expression of genes involved in synaptic pathways related to glutamate, gamma-amino butyric acid (GABA), and dopamine signaling in murine NSC-34 motor-neuron-like cells [10], which is a cell line used as a model of motor neuron development [11].

However, nowadays, “minor phytocannabinoids” are also receiving attention. A precise definition of minor phytocannabinoids is missing, but this term is used to indicate those phytocannabinoids different from the “Big Four” whose biological profile is still poorly characterized [12]. Recently, our group has investigated the effects of some minor cannabinoids, such as Δ^8^-tetrahydrocannabinol (Δ^8^-THC) and the less-known cannabinerol (CBNR), showing their role in neuronal differentiation [13,14]. In differentiated SH-SY5Y cells, ∆^8^-THC at the concentration of 20 µM resulted in the upregulation of the expression of genes involved in the glutamatergic pathway and the inhibition of the expression of genes in the cholinergic one. Conversely, ∆^8^-THC did not affect the expression of genes involved in the GABAergic and dopaminergic pathways. In addition, CBNR was tested in a proliferative NSC-34 cell line at different concentrations (from 5 to 50 µM), showing no toxicity and the capacity to regulate some differentiative neuronal genes, such as *Map2* and *Rock1*. Moreover, the phosphorylation of the AKT protein, a key mediator of the neuronal survival pathway, reaches its peak at 10 µM. The serine/threonine protein kinase AKT, a downstream effector of phosphoinositide 3-kinase (PI3K), is known to play a crucial role in different physiological functions. It activates Cyclic AMP-Responsive Element-Binding Protein 1 (CREB1), which is a transcription factor that binds to the cAMP response element (CRE) of the promoters of its target genes. Its activation leads to the expression of genes involved in cell survival, differentiation, and, especially in neurons, synaptic plasticity [15,16].

In this study, we focused our attention on CBNR, which is a geometric isomer of CBG that was discovered thanks to the isolation of its precursor, cannabinerolic acid, from air-dried cannabis leaves [17,18]. It was chemically identified in 2016 from an Indian hashish sample [19]. Since not much information is available about the roles of CBNR in nervous system development, we addressed this point by analyzing the transcriptomic profile of undifferentiated NSC-34 motor-neuron-like cells treated with 10 µM CBNR via next-generation sequencing analysis.

## 2. Materials and Methods

### 2.1. Cell Culture and Treatment

**Figure 1:**
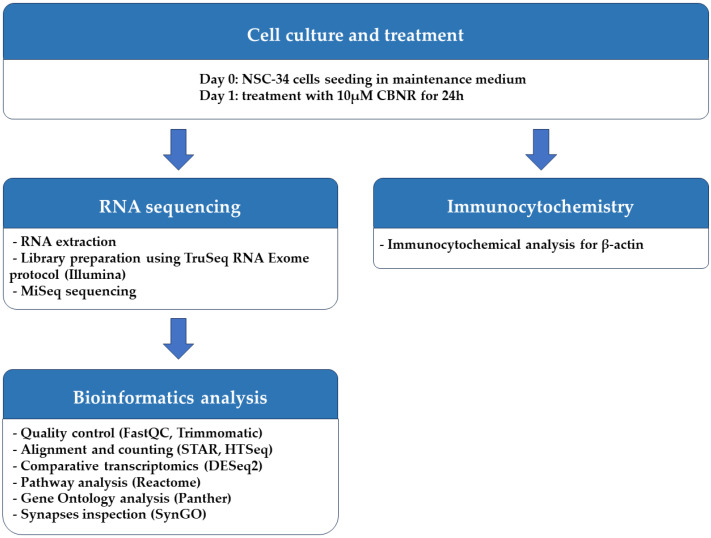
Workflow of methods used.

The NSC-34 motor-neuron-like cell line was obtained from Cellutions Biosystems Inc., Cedarlane (Burlington, ON, Canada). NSC-34 cells were plated onto 6-well plates (#130184, Thermo Scientific, Rochester, NY, USA) at the density of 500,000/well or on coverslips (12 mm; Epredia, Braunschweig, Germany) at the density 100,000/well in 24-well plates (#3526, Corning Incorporated, New York, NY, USA) in DMEM-high glucose medium (#D5671, Sigma-Aldrich, Saint Louis, MO, USA) supplemented with 10% fetal bovine serum (FBS) (#F7524, Sigma-Aldrich, Saint Louis, MO, USA), 1% penicillin/streptomycin (#P0781, Sigma-Aldrich, Saint Louis, MO, USA) and 1% L-glutamine (#G7513, Sigma-Aldrich, Saint Louis, MO, USA) at 37 °C in a moisturized atmosphere of 5% CO_2_ and 95% air. Then, 24 h after plating, NSC-34 cells were treated for 24 h with 10 µM CBNR (provided by the Department of Pharmaceutical Sciences, University of Eastern Piedmont). CBNR was dissolved in dimethylsulfoxide (DMSO) (#D8418, Sigma-Aldrich, Saint Louis, MO, USA) and further diluted in phosphate-buffered saline (PBS) 1× (#806552, Sigma-Aldrich, Saint Louis, MO, USA). The final DMSO concentration was <0.1 In a previous study, we have already shown that CBNR is not cytotoxic at the concentration of 10 µM, using the MTT assay [14]. At the end of the treatment, cells were further processed for RNA extraction or immunocytochemistry as reported in the following paragraphs. All the steps executed to perform the experiment are resumed in Figure 1.

### 2.2. RNA Extraction and Library Preparation

At the end of the treatment, cells were harvested using 0.25% trypsin–ethylenediaminetetraacetic acid (EDTA) solution (#T4049, Sigma-Aldrich, Saint Louis, MO, USA) and pelleted by centrifugation (300× *g* for 5 min). Then, the pellet was processed for RNA extraction. Total RNA was extracted using the Maxwell^®^ RSC simplyRNA Cells Kit (#AS1390, Promega, Madison, WI, USA) with the Maxwell^®^ RSC instrument. Library preparation of two biological replicates was carried out with TruSeq^®^ RNA Exome protocol (#20020492, #20020189, #20020490, #20020183, Illumina, San Diego, CA, USA) as already reported [10], following the manufacturer’s instructions. Briefly, 100 ng of total RNA was fragmented in a thermal cycler for 8 min at 94 °C, which was followed by cDNA first-strand synthesis using the reverse transcriptase SuperScript II (#18064-014, Invitrogen, Carlsbad, CA, USA). Subsequently, the second-strand synthesis was carried out to obtain the double-stranded cDNA, which was followed by a purification step using AMPure XP beads (#63881, Beckman Coulter Inc., Brea, CA, USA). Fragment adenylation was performed at 3′ ends to allow the ligation of the complementary adapters, thereby avoiding the development of large chimeras. Then, Adapter-Indexes (TruSeq^®^ RNA Single Indexes Set A, #20020492, Illumina, San Diego, CA, USA) were ligated to the cDNA fragments, and after a purification step, a PCR amplification was performed using the primer provided by TruSeq^®^ RNA Library Prep for Enrichment #20020189 (denaturation: 30 s at 98 °C; 15 cycles: 10 s at 98 °C, 30 s at 60 °C, 30 s at 72 °C; extension: 5 min at 72 °C). The regions of interest were selected and enriched using the TruSeq^®^ RNA Enrichment (#20020490, Illumina, San Diego, CA, USA) through a first reaction of hybridization, where the exome capture probes (Illumina Exome Panel—Enrichment Oligos Only, #20020183, Illumina, San Diego, CA, USA) were mixed with the cDNA library (200 ng of each library). The pool was purified by streptavidin-conjugated magnetic beads. Then, a second hybridization and purification were performed. Afterwards, second PCR amplification was performed. The Tapestation 4150 instrument (Agilent, Richardson, TX, USA), using the D1000 screentape (#5067-5582 and #5067-5583; Agilent, Richardson, TX, USA), was used to validate the quality of the library. A denaturation step using 0.2N sodium hydroxide (NaOH) was performed, and then it was diluted until it reached a concentration of 13 pM. The MiSeq Reagent Kit v3 (600 cycles) (#MS-102-3003, Illumina, San Diego, CA, USA) was used for sequencing with the Illumina instrument MiSeq (Illumina, San Diego, CA, USA). The run was made in paired-end mode.

### 2.3. Bioinformatics Analysis

The quality of the runs was confirmed by FastQC version 0.11.9 (Babraham Institute, Cambridge, UK) and the bases with low quality score, and adapters were removed by Trimmomatic version 0.40-rc1 (Usadel Lab, Aachen, Germany) [20]. The reads were then mapped against the vM28 genome from gencode using the STAR RNA-seq aligner version 2.7.10a_alpha_220207 (New York, NY, USA) [21]. The raw counts were computed using HTSeq at version 0.13.5 [22] for each gene included in the vM28 reference genome. Comparative transcriptomics were performed using the DESeq2 library version 1.36.0 [23] in R version 4.2.0 (R Core Team). Corrected *p*-values, defined as q-values, were computed using the Benjamini–Hochberg method, and a threshold of 0.05 was set to remove false-positive genes. Additionally, the analysis was corrected for batch effects. Genes were defined as differentially expressed genes (DEGs) when the associated q-value was lower than 0.05. The “Analysis gene list” tool of Reactome [24] was used to observe overrepresented DEGs for pathways using default parameters. Pathways were considered significantly overrepresented if the False Discovery Rate (FDR) was lower than 0.05. We then identified cellular component terms that overrepresented our DEGs using the Panther (protein annotation through evolutionary relationship) website [25]. The analysis was conducted against DEGs included in the “Nervous system development” R-HSA-9675108 pathway compared to our full list of DEGs as the background with default parameters. Since the pivotal role was identified by terms associated with synapse, we took advantage of the SynGO database [26] in which we characterized the full list of our DEGs with default parameters. Both scatter plots and barplots were generated in R using libraries from the Bioconductor project with the “BiocManager” library version at 1.30.20. Specifically, we used the “ggplot2” library at version 3.4.2 and “gridExtra” at version 2.3. To easily manipulate data, we took advantage of “readxl”, “dplyr” and “stringr” with versions 1.4.2, 1.1.0 and 1.5.0, respectively.

### 2.4. Immunocytochemistry

At the end of the treatment, cells were fixed for 20 min with 4% paraformaldehyde (sc-281692, Santa Cruz Biotechnologies, Dallas, TX, USA) at room temperature (RT). Next, cells were washed three times for 5 min with PBS and incubated with 3% H_2_O_2_ (#H1009, Sigma-Aldrich, Saint Louis, MO, USA) at RT for 30 min in order to suppress the endogenous peroxidase activity. After three washes of 5 min with PBS, cells were processed according to the Vectastain^®^ Elite^®^ ABC-HRP kit (#PK-6200, Vectastain, Glostrup, Denmark) instructions. Briefly, cells were blocked with horse serum for 20 min at RT. Then, they were incubated at 4 °C overnight with the anti-β-Actin antibody (1:100; #sc-47778, Santa Cruz Biotechnologies, Dallas, TX, USA). Afterward, cells were washed three times for 5 min with PBS 1× and incubated for 30 min at RT with biotinylated secondary antibody. After three washes with PBS, cells were incubated with streptavidin AB complex–horseradish peroxidase (HRP) for 30 min at RT. The immunostaining was developed with the 3,3′-Diaminobenzidine (DAB) peroxidase substrate kit (#SK-4100, Vector Laboratories, Newark, CA, USA) and counterstained with nuclear fast red (#H-3403, Vector Laboratories, Newark, CA, USA). The images were captured using a light microscopy (Axioscope 5 combined with axiocam 208 color camera; Zeiss, Oberkochen, Germany) equipped with an oil immersion objective 100× planabo.

## 3. Results

### 3.1. CBNR Modulates Synaptic Genes

**Figure 2:**
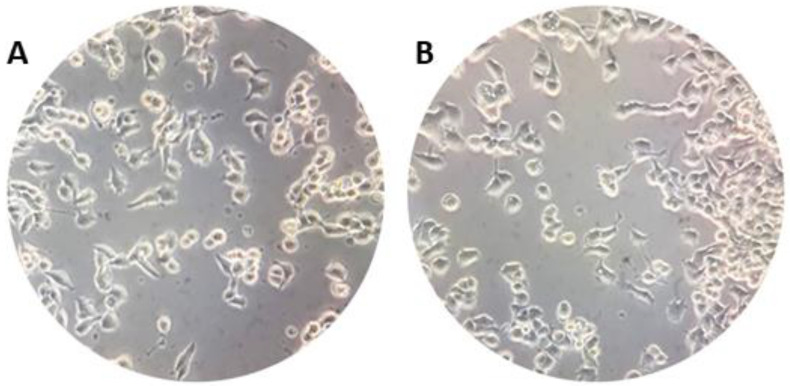
(**A**) NSC-34 control cells and (**B**) 10 µM CBNR-treated cells. Objective 40×.

In this study, NSC-34 motor-neuron-like cells were treated with CBNR at a concentration of 10 µM (CBNR10 group) for 24 h. At the end of the treatment, both treated and untreated NSC-34 cells (CTRL group), shown in Figure 2, were harvested for RNA extraction, and the transcriptomic profile was obtained using next-generation sequencing technologies.

**Figure 3:**
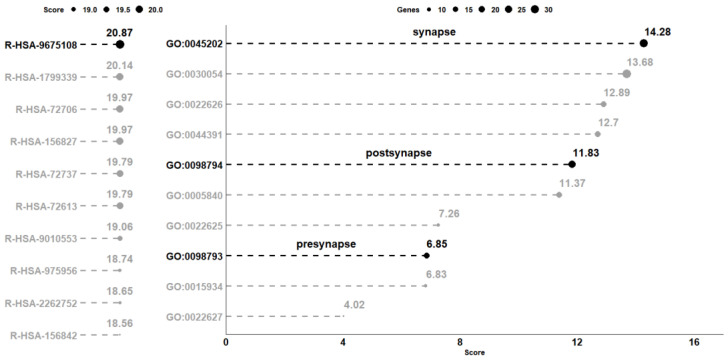
Analysis of the overrepresented pathway and gene ontology terms in the comparison CTRL vs. CBNR10. On the left panel, the 10 pathways collected in the Reactome database with the highest score computed as log_2_(FDR) are highlighted. Conversely, on the right part of the figure, the 10 most overrepresented cellular component terms obtained with Panther, using the DEGs included in the R-HSA-9675108 (“Nervous System Development”) pathway, are shown. The score, computed in the same way as the one computed for the pathways, is plotted on the x-axis. For terms, the size of each point is representative of the number of DEGs included in the term. All score values are rounded to the second digit.

The comparative analysis between CTRL and CBNR10 revealed 685 DEGs. The complete list of DEGs is included in Table S1. To understand the biological effects of CBNR, we examined the pathways involving the DEGs using the Reactome database (accessed on 20 October 2023). Reactome is an open-source, open-access, manually curated, peer-reviewed pathway database. In particular, the “Analysis gene list” tool of Reactome highlighted the presence of 98 overrepresented pathways, which were collected in Table S2. Notably, among the most significant pathways identified by Reactome, “Nervous system development” (R-HSA-9675108) had the highest number of entities found (71 out of 621, with 62 characterized as genes). The 10 pathways with the highest score, computed as log_2_(FDR), were plotted in the left panel of Figure 3. Thus, we focused on the 62 DEGs included in the “Nervous system development” (R-HSA-9675108) term and, to better understand the role of these DEGs modulated by CBNR, we inspected the subcellular structure or localization in which these genes exert their functions. In this regard, we searched for the overrepresented cellular component terms included in the “Nervous system development” using Panther (http://www.pantherdb.org/, accessed on 10 November 2023). The Panther (protein annotation through evolutionary relationship) classification system combines gene function, ontology, pathways, and statistical analysis tools for gene expression experiments. We found 20 overrepresented terms, most of which are related to synapses and ribosomes. The 10 most overrepresented cellular component terms are displayed in the right panel of Figure 3. Additionally, all the details of the overrepresentation analysis performed by Panther are presented in Table S3. Interestingly, among the cellular component terms, “synapse” (GO:0045202), “postsynapse” (GO:0098794), and “presynapse” (GO:0098793) have very high scores (14.28, 11.83 and 6.85, respectively).

**Figure 4:**
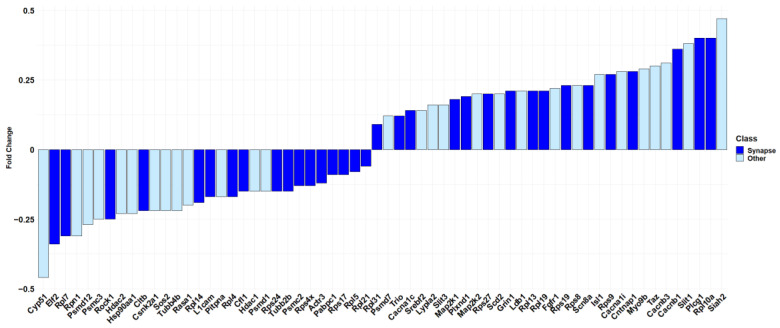
Barplot showing the fold change in the “Nervous system development” pathway of Reactome. In dark blue, the 33 DEGs of the Reactome pathway that are also included in the “synapse” cellular component term overrepresented in Panther are highlighted. In light blue, the DEGs of the Reactome pathway excluded from “synapse” cellular component term over-represented in Panther. The fold change scale is representative of the log_2_(CBNR10/CTRL).

Specifically, “synapse” has the highest score among all the other terms (log_2_(FDR) = 14.28). It is important to note, as shown in Figure 4, that more than half of the genes included in “Nervous system development” (R-HSA-9675108) are also part of “synapse” (GO:0045202) (33 out of 62 genes).

### 3.2. CBNR Modulates Genes Associated with Cytoskeleton and Ion Channels

**Figure 5:**
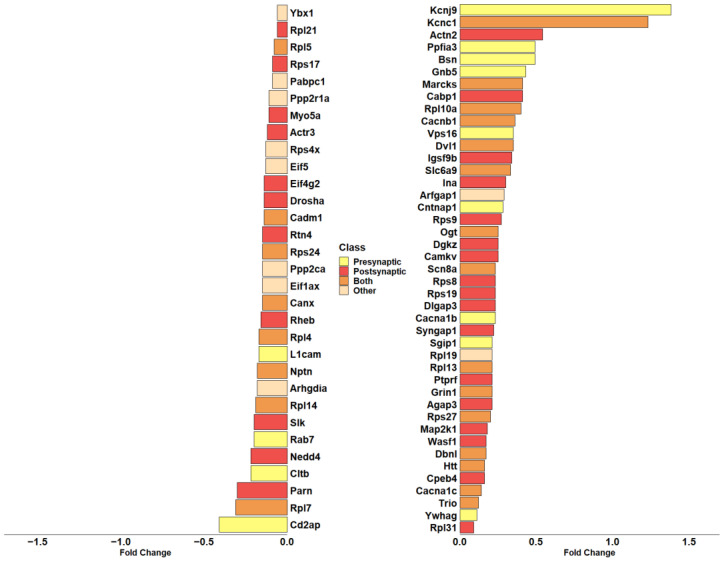
Barplot showing the fold change in DEGs included in the overrepresented “synapse” term of cellular component in SynGo. The color of each bar identifies the cellular compartment. DEGs included in the “Presynapse” term are indicated in yellow. The red color indicates DEGs belonging exclusively to the “Postsynapse” term. Dark orange indicates the DEGs present in both “Presynapse” and “Postsynapse” terms. The DEGs not included in “Presynapse” or “Postsynapse” are presented in light orange. The fold change scale represents the log_2_(CBNR10/CTRL).

Given the high representation of synaptic genes in our analysis, we evaluated the role of DEGs modulated by CBNR in the synapse, taking advantage of the SynGo database (accessed on 15 November 2023). SynGo is an evidence-based, manually expert-curated database for synapse gene ontology. Therefore, we first mapped our list of 685 DEGs using the “ID convert tool”, which correctly converted 682 DEGs from *Homo sapiens* to *Mus musculus*. Then, with the default parameters, we performed the “geneset analysis” against a background including the “brain expressed” genes. The analysis found 86 unique SynGO annotated genes and 92 overrepresented cellular component terms, as shown in Table S4. Among them, 74 DEGs are annotated with the overrepresented cellular component terms, and “synapse” (GO:0045202) is still the most general parent term. Additionally, as shown by SynGo itself, “presynapse” (GO:0098793) and “postsynapse” (GO:0098794) terms are overrepresented, as depicted in Figure S1. Thus, we highlighted all DEGs included in “synapse” with a focus on pre- or post-synaptic localization. In this sense, Figure 5 includes all DEGs part of “synapse” and their classification as presynapse and/or postsynapse genes.

Interestingly, most of them (28 DEGs) are included in postsynapse (*Actn2*, *Actr3*, *Agap3*, *Cabp1*, *Camkv*, *Cpeb4*, *Dgkz*, *Dlgap3*, *Drosha*, *Eif4g2*, *Igsf9b*, *Ina*, *Map2k1*, *Myo5a*, *Nedd4*, *Parn*, *Ptprf*, *Rheb*, *Rpl21*, *Rpl31*, *Rps17*, *Rps19*, *Rps8*, *Rps9*, *Rtn4*, *Slk*, *Syngap1*, *Wasf1*). Then, 13 DEGs are only part of presynapse (*Bsn*, *Cacna1b*, *Cd2ap*, *Cltb*, *Cntnap1*, *Gnb5*, *Kcnj9*, *L1cam*, *Ppfia3*, *Rab7*, *Sgip1*, *Vps16*, *Ywhag*), while 23 DEGs play a role both in presynaptic and postsynaptic compartments (*Cacna1c*, *Cacnb1*, *Cadm1*, *Canx*, *Dbnl*, *Dvl1*, *Grin1*, *Htt*, *Kcnc1*, *Marcks*, *Nptn*, *Ogt*, *Rpl10a*, *Rpl13*, *Rpl14*, *Rpl4*, *Rpl5*, *Rpl7*, *Rps24*, *Rps27*, *Scn8a*, *Slc6a9*, *Trio*). Only 10 DEGs (*Arfgap1*, *Arhgdia*, *Eif1ax*, *Eif5*, *Pabpc1*, *Ppp2ca*, *Ppp2r1a*, *Rpl19*, *Rps4x*, *Ybx1*) do not have a specific participation in pre- or post-synaptic compartments and are then classified as “Other” in Figure 5.

We annotated the found DEGs in terms of biological processes via SynGO, as shown in Table 1. The main categories that resulted were related to cytoskeleton, translation, synapse organization, and synaptic transmission.

**Figure 6:**
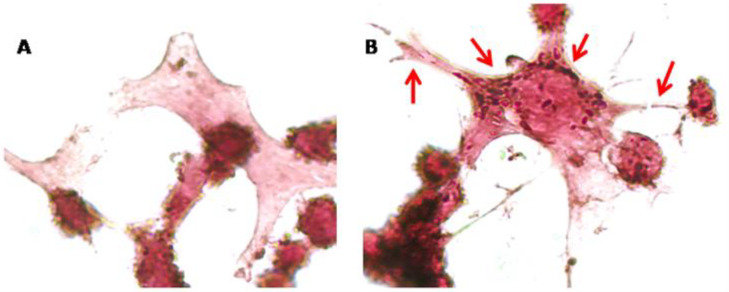
Immunocytochemical analysis of NSC-34 cells. Control cells (**A**) and 10 µM CBNR treated cells (**B**) were immunoprofiled for β-actin. Objective: 100×. Red arrows indicate positive staining for β-actin.

Finally, since CBNR influences the expression of synaptic genes related to the cytoskeleton, we examined cytoskeletal changes in NSC-34 cells exposed to 10 µM CBNR for 24 h. In this regard, we performed immunocytochemical analysis for β-actin. Figure 6 shows that CBNR treatment increased β-actin staining and localization in dendritic spines and cytoplasm. This β-actin expression supports the transcriptomic results about cytoskeletal dynamics. Indeed, β-actin is enriched in dynamic neuronal structures such as dendritic spines as development progresses, supporting that β-actin is the major actin isoform fueling actin dynamics [27].

## 4. Discussion

In the last few years, cannabinoids have received great attention as potential drugs for the treatment of neurological disorders. Common features of these pathologies include impairments in cognitive and motor functions [1,2], which may be attributed to neuronal inactivity, cell death, or synapse dysfunctions [28]. For this reason, neuroprotective strategies capable of modulating synaptogenesis and synaptic transmission are needed. Among cannabinoids, CBD and CBG have been extensively investigated in rodents and have already been shown to improve behavioral and motor functions in vivo and to regulate synaptic pathways in vitro on NSC-34 motor-neuron-like cell line [4,10]. Interestingly, CBG and CBD have also shown neuroprotective potential in an in vitro model of neuroinflammation and a model of injured NSC-34 motor-neuron cells [29,30,31]. The NSC-34 cell line is obtained by the hybridization of an embryonic spinal cord and neuroblastoma cells from mice. This cell line is mainly enriched with motor neurons and thus is widely used as a model for a developing motor neuron system [11]. Motor neurons play a fundamental role in the neuromuscular junction (NMJ), and their degeneration causes the typical NMJ impairment that characterizes some neurological diseases [32]. For example, the impairment of synaptic vesicle release due to a defect of calcium influx causes the Lambert–Eaton myasthenic syndrome, whereas the acquired neuromyotonia is characterized by the downregulation of voltage-gated potassium channels, leading to a prolonged depolarization of the nerve terminal. Finally, defects in NMJ formation and maintenance are peculiar to myasthenia gravis and congenital myasthenia syndrome [33].

Recently, minor cannabinoids, such as Δ^8^-THC and CBNR, have also been studied to elucidate whether they exhibit similar neuroprotective properties compared to major ones [13,14]. In this regard, Δ^8^-THC protected retinoic acid-differentiated neuroblastoma SH-SY5Y cells against amyloid beta (Aβ) toxicity. In particular, Δ^8^-THC pre-treatment restored the loss of cell viability and was able to modulate biological processes related to ER functions and proteostasis. Δ^8^-THC pre-treatment counteracted the Aβ-induced upregulation of genes involved in ER stress and unfolded protein response. As a consequence, apoptosis was reduced. These results suggested that Δ^8^-THC may be a new neuroprotective strategy against amyloid toxicity [34]. However, also other cannabinoids, such as cannabidiolic acid (CBDA) and tetrahydrocannabinolic acid (THCA), have also been shown to reduce Aβ and tau pathology in a murine Alzheimer’s disease model [35]. CBD showed protective effects in in vitro and in vivo Alzheimer’s disease models [36,37]. In addition, a study evidenced that THC molecules can bind and disrupt the amyloid protofibril structure [38].

In this study, we focused our attention on CBNR, a geometric isomer of CBG, that we have previously tested for its involvement in neuronal differentiation [14]. Here, we investigated the impact of CBNR on the physiology of NSC-34 motor-neuron-like cells and its effects on the regulation of synaptic-related genes. We used NSC-34 cells in an undifferentiated state in order to understand whether, during an early developmental stage, the CBNR can influence important processes, such as synaptogenesis, which is peculiar then of post-mitotic motor neurons. Specifically, NSC-34 cells were treated for 24 h with 10 µM CBNR, which is a concentration previously reported to be not cytotoxic [14]. We performed a next-generation sequencing analysis, resulting in 685 genes deregulated by CBNR (Table S1). Interestingly, using the Reactome database, we found that the most significant overrepresented pathway was “Nervous system development”, which included 62 genes. Gene ontology analysis of the cellular component using Panther revealed that more than half of the 62 DEGs belong to synapse localization. These results suggest that CBNR influences synaptic genes (Figure 3 and Figure 4). In order to assess all synaptic genes regulated by CBNR, we analyzed our 685 DEGs with the SynGO database. Results showed that “synapse” is the highest level overrepresented term with 74 genes along with children-levels “postsynapse” (51 genes) and “presynapse” (36 genes) terms (Figure 5, Table S4, Figure S1). Remarkably, the comparison with different CBNR doses (Figure S2) showed that the expression of these synaptic genes is not dose-dependent, supporting that the concentration of 10 µM CBNR affects mainly synaptic processes. Surprisingly, terms related to ribosome structure were also overrepresented (Table S4). However, ribosomal genes did not show a clear trend, since many of them were both down- and upregulated (Figure 5).

**Figure 7:**
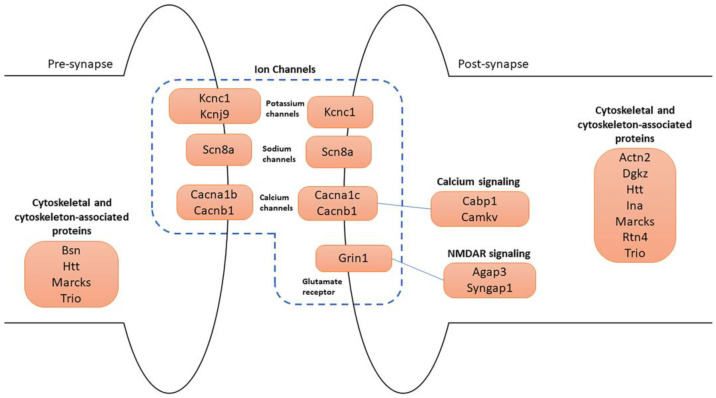
Schematic representation of the main synaptic DEGs. Four major categories of genes are illustrated in pre- and post-synapse: (1) ion channels, which include potassium, sodium, calcium channels, and glutamate receptor; (2) calcium signaling; (3) NMDAR signaling; (4) cytoskeletal and cytoskeleton-associated protein-coding genes.

Many of the 74 synaptic genes are involved in synapse organization and specialization, cytoskeleton, and ion channels (Figure 7).

Cytoskeletal and cytoskeleton-associated proteins play a fundamental role in neuronal development and physiology. *Trio* emerged as a central regulator of actin dynamics at the synapse and has a role in neurite growth and axon guidance. At the presynapse, *Trio* regulates the actin dynamics, facilitating vesicle release and trafficking. Postsynaptically, *Trio* has a role in synaptic plasticity and was found in postsynaptic density (PSD) [39]. TRIO is targeted to presynaptic terminals thanks to its association with the BASSOON protein, encoded by the *Bsn* gene, which is also upregulated in our study [40]. At the presynaptic level, BASSOON acts as a presynaptic scaffold required for neurotransmission, and it controls synaptic vesicle release [41]. Bassoon is also important for synapse integrity [42]. Other proteins involved in the regulation of the actin cytoskeleton are α-ACTININ-2 and MARCKS, encoded by *Actn2* and *Marcks* genes, respectively, which resulted in both being upregulated. α-ACTININ-2 modulates spine maturation to mediate synaptogenesis [43]. MARCKS has been implicated in dendrite morphology [44].

Of note, we observed the downregulation of the *Rtn4* gene. NOGO-A represents the largest splice form of this gene. Neuronal NOGO-A is known to negatively regulate dendritic morphology and synaptic transmission and to reduce axon growth and regeneration [45,46].

The HUNTINGTIN protein, encoded by the *Htt* gene, is also present at both pre- and post-synapses. Presynaptically, *Htt* positively regulates endocytosis, exocytosis, and axonal transport, and it is a binding partner of BASSOON [47]. Postsynaptically, it regulates dendritic transport and receptor localization [48]. The *Dgkz* gene encodes for DGKζ, a DGK isoform enriched at excitatory synapses, which is shown to be able to regulate spine density in cultured neurons. Specifically, DGKζ deficiency in mice causes a reduction in spine density and excitatory synaptic transmission [49].

In support of our transcriptomic results, the immunocytochemistry for β-actin of NSC-34 cells treated with 10 µM CBNR evidenced cytoskeletal changes (Figure 6).

These results suggest that CBNR can activate in NSC-34 motor neuron cells a transcriptional program that reorganizes the cytoskeletal organization of pre- and post-synapse. Through the modulation of these genes, the correct formation and maintenance of synapses can be regulated. In this context, scaffold proteins, such as BASSOON, can allow the correct localization of other presynaptic proteins. The modulation of cytoskeletal-related genes is also an interesting result considering the pivotal role played by cytoskeletal organization in synaptic vesicle transport.

Remarkably, genes encoding for calcium (Ca^2+^), sodium (Na^+^), and potassium (K^+^) ion channel components were all upregulated. These ions play a fundamental role in neuronal functions. Indeed, ion gradients represent a form of intra- and inter-cellular communication within networks of neurons. Specifically, Ca^2+^ signaling has a role in the release of neurotransmitters and the modulation of synaptic plasticity. Na^+^ entry in neurons mediates the initiation and propagation of action potentials, while the efflux of potassium ions participates in the repolarization of membrane potential after depolarization [50]. *Cacna1b*, *Cacna1c*, and *Cacnb1* genes encode for calcium voltage-gated channel subunits, which mediate the influx of calcium ions into the cell upon membrane polarization [51,52]. They regulate intracellular processes such as contraction, secretion, neurotransmission, and gene expression. Specifically, CACNA1B, expressed at the presynaptic level, is the pore-forming subunit of an N-type voltage-dependent calcium channel, which controls neurotransmitter release from neurons. CACNA1C and CACNB1 are pore-forming and regulatory subunits of L-type voltage-dependent calcium channels, respectively. Then, the *Grin1* gene encodes the main subunit of the ionotropic N-methyl-D-aspartate (NMDA)-type glutamate receptor, which is a key component of glutamatergic synapses. The NMDA receptor is permeable to Ca^2+^ and Na^+^ ions, increases excitatory postsynaptic potentials (EPSPs), and triggers downstream signaling pathways involved in the regulation of many physiological and pathophysiological processes [53]. Another gene related to Na^+^ permeability is *Scn8a*, which encodes a pore-forming subunit of a voltage-gated sodium channel. It is essential for the rapid membrane depolarization that occurs during the generation of the action potential in neurons. Mutations in this gene are associated with cognitive disability and epileptic encephalopathy [54].

Regarding potassium flux, *Kcnc1* and *Kcnj9* were highly upregulated. The first encodes a protein that mediates the voltage-dependent potassium channel permeability, causing the rapid repolarization of neurons (i.e., outflux of potassium ions). *Kcnc1*-related disorders include a form of epilepsy and ataxia [55]. The second gene encodes a G-protein-activated inward-rectifier channel (GIRK) protein expressed at the presynaptic level. These channels allow potassium to flow into the cell rather than out of it. Many studies suggest a critical involvement of *Kcnj9* in neurological and psychiatric conditions [56].

Moreover, two calcium effectors were also upregulated. *Cabp1* gene encodes a calcium-binding protein that shares similarities with calmodulin. It regulates the gating of voltage-gated calcium ion channels [57]. The *Camkv* gene, encoding for a pseudokinase, is involved in the maintenance of the dendritic spine and contributes to normal synaptic transmission in a Ca^2+^/calmodulin-dependent manner [58].

Finally, *Agap3* and *Syngap1*, which we found upregulated in our analysis, are both essential components of the NMDA receptor signaling complex at the PSD level of excitatory synapses. The AGAP3 protein mediates long-term potentiation in synapses by linking activation of the NMDA receptor to alpha-amino-3-hydroxy-5-methyl-4-isoxazolepropionic acid (AMPA) receptor trafficking [59]. *Syngap1*, encoding the synaptic Ras GTPase-activating protein, plays a central role in synaptic plasticity. It is a major genetic risk factor for global developmental delay, intellectual disability, autism spectrum disorder, and epileptic encephalopathy [60,61].

Interestingly, this is the first study investigating the role of CBNR in synaptic transmission, contributing to deep knowledge about the effects of this emerging cannabinoid on neuronal cells.

However, we have also to highlight some limitations of this study. First, these results should be reconfirmed in a more physiological model with respect to a cell line, such as induced pluripotent stem cells [62], olfactory neuroepithelial cells [63], or transdifferentiation of human circulating monocytes [64]. Moreover, we did not compare our results with a pro-differentiative compound.

## 5. Conclusions

In this study, we discovered that the phytocannabinoid CBNR can regulate the genes associated with cytoskeleton organization and ion channels of the synapse in NSC-34 motor-neuron-like cells. Thus, these findings suggest a role of CBNR in the formation of the synapse and synaptic transmission. Considering these results, CBNR may be tested in a neurological disease model to investigate a potential neuroprotective role. Therefore, our work paves the way for further in vitro and in vivo studies required to assay the effects of this phytocannabinoid in the treatment of synaptic dysfunctions that characterize motor disabilities in many neurological disorders.

## Figures and Tables

**Table 1:** Biological process terms associated with DEGs involved in the synapse in SynGO.

Gene	Fold Change	Biological Process
*Actn2*	0.54	structural constituent of postsynaptic actin cytoskeleton
*Actr3*	−0.12	postsynapse organization; postsynaptic actin cytoskeleton organization
*Agap3*	0.21	regulation of postsynaptic membrane neurotransmitter receptor levels
*Arfgap1*	0.29	-
*Arhgdia*	−0.18	regulation of synaptic vesicle cycle
*Bsn*	0.49	regulation of synaptic vesicle cycle; structural constituent of active zone; synaptic vesicle clustering
*Cabp1*	0.41	modification of postsynaptic actin cytoskeleton; postsynapse to nucleus signaling pathway
*Cacna1b*	0.23	regulation of calcium-dependent activation of synaptic vesicle fusion; voltage-gated calcium channel activity involved in regulation of presynaptic cytosolic calcium levels
*Cacna1c*	0.14	-
*Cacnb1*	0.36	-
*Cadm1*	−0.14	maintenance of postsynaptic specialization structure; presynapse assembly; retrograde trans-synaptic signaling by trans-synaptic protein complex; synapse adhesion between pre- and postsynapse; synapse assembly
*Camkv*	0.25	modulation of chemical synaptic transmission; regulation of modification of postsynaptic structure
*Canx*	−0.15	synaptic vesicle endocytosis
*Cd2ap*	−0.41	synapse organization
*Cltb*	−0.22	synaptic vesicle endocytosis
*Cntnap1*	0.28	regulation of synapse maturation
*Cpeb4*	0.16	-
*Dbnl*	0.17	postsynaptic actin cytoskeleton organization; structural constituent of postsynaptic actin cytoskeleton
*Dgkz*	0.25	maintenance of postsynaptic density structure
*Dlgap3*	0.23	modification of postsynaptic structure; modulation of chemical synaptic transmission
*Drosha*	−0.14	postsynaptic density
*Dvl1*	0.35	postsynapse organization; presynapse assembly; regulation of postsynapse organization; regulation of synaptic vesicle exocytosis
*Eif1ax*	−0.15	-
*Eif4g2*	−0.14	-
*Eif5*	−0.13	-
*Gnb5*	0.43	-
*Grin1*	0.21	ligand-gated ion channel activity involved in regulation of presynaptic membrane potential; transmitter-gated ion channel activity involved in regulation of postsynaptic membrane potential
*Htt*	0.16	postsynapse to nucleus signaling pathway
*Igsf9b*	0.34	synapse adhesion between pre- and postsynapse
*Ina*	0.30	postsynaptic modulation of chemical synaptic transmission; structural constituent of postsynaptic intermediate filament cytoskeleton
*Kcnc1*	1.23	voltage-gated ion channel activity involved in regulation of presynaptic membrane potential
*Kcnj9*	1.38	regulation of presynaptic membrane potential
*L1cam*	−0.17	modulation of chemical synaptic transmission; regulation of synapse assembly
*Map2k1*	0.18	regulation of neurotransmitter receptor localization to postsynaptic specialization membrane
*Marcks*	0.41	regulation of modification of postsynaptic actin cytoskeleton
*Myo5a*	−0.11	establishment of endoplasmic reticulum localization to postsynapse; regulation of postsynaptic cytosolic calcium levels; transport
*Nedd4*	−0.22	regulation of postsynaptic neurotransmitter receptor endocytosis; regulation of protein catabolic process at postsynapse, modulating synaptic transmission; regulation protein catabolic process at postsynapse
*Nptn*	−0.18	trans-synaptic signaling by trans-synaptic complex, modulating synaptic transmission
*Ogt*	0.25	regulation of neurotransmitter receptor localization to postsynaptic specialization membrane; regulation of synapse assembly
*Pabpc1*	−0.09	-
*Parn*	−0.30	-
*Ppfia3*	0.49	presynapse assembly; synaptic vesicle docking; synaptic vesicle exocytosis
*Ppp2ca*	−0.15	-
*Ppp2r1a*	−0.11	-
*Ptprf*	0.21	regulation of postsynapse organization; synapse adhesion between pre- and postsynapse;
*Rab7*	−0.20	neurotransmitter receptor transport, postsynaptic endosome to lysosome; synaptic vesicle recycling via endosome
*Rheb*	−0.16	regulation of postsynapse organization
*Rpl10a*	0.40	translation at postsynapse: translation at presynapse
*Rpl13*	0.21	translation at postsynapse; translation at presynapse
*Rpl14*	−0.19	translation at postsynapse; translation at presynapse
*Rpl19*	0.21	-
*Rpl21*	−0.06	-
*Rpl31*	0.09	-
*Rpl4*	−0.17	translation at postsynapse; translation at presynapse
*Rpl5*	−0.08	translation at postsynapse; translation at presynapse
*Rpl7*	−0.31	translation at postsynapse; translation at presynapse
*Rps17*	−0.09	-
*Rps19*	0.23	-
*Rps24*	−0.15	translation at postsynapse; translation at presynapse
*Rps27*	0.20	translation at postsynapse; translation at presynapse
*Rps4x*	−0.13	-
*Rps8*	0.23	-
*Rps9*	0.27	-
*Rtn4*	−0.15	modulation of chemical synaptic transmission; regulation of postsynapse assembly
*Scn8a*	0.23	-
*Sgip1*	0.21	synaptic vesicle endocytosis
*Slc6a9*	0.33	neurotransmitter uptake
*Slk*	−0.20	modulation of chemical synaptic transmission
*Syngap1*	0.22	maintenance of postsynaptic specialization structure; modulation of chemical synaptic transmission
*Trio*	0.12	modulation of chemical synaptic transmission; postsynaptic modulation of chemical synaptic transmission
*Vps16*	0.35	-
*Wasf1*	0.17	dendritic transport of mitochondrion; modification of postsynaptic actin cytoskeleton
*Ybx1*	−0.06	-
*Ywhag*	0.11	-

The fold change is computed as log_2_(CBNR10/CTRL). All the values are rounded to the second decimal digit.

## Data Availability

The data presented in this study are openly available in the NCBI Sequence Read Archive at BioProject, accession numbers PRJNA839187 and PRJNA1043513.

## Supplementary Materials

The following supporting information can be downloaded at https://www.mdpi.com/article/10.3390/biomedicines12010189/s1, Table S1: DEGs resulted in CTRL vs. CBNR10; Table S2: Overrepresented pathway in Reactome; Table S3: Overrepresented cellular component terms in Panther; Table S4: Overrepresented gene ontology terms in SynGO; Figure S1: SynGO diagram of overrepresented synaptic ontology terms; Figure S2: Heatmap of synaptic DEGs at different doses of CBNR.

## References

[1] Ding C., Wu Y., Chen X., Chen Y., Wu Z., Lin Z., Kang D., Fang W., Chen F. (2022). Global, regional, and national burden and attributable risk factors of neurological disorders: The Global Burden of Disease study 1990–2019. Front. Public Health.

[2] Van Schependom J., D’Haeseleer M. (2023). Advances in Neurodegenerative Diseases. J. Clin. Med..

[3] Dejanovic B., Sheng M., Hanson J.E. (2024). Targeting synapse function and loss for treatment of neurodegenerative diseases. Nat. Rev. Drug Discov..

[4] Stone N.L., Murphy A.J., England T.J., O’Sullivan S.E. (2020). A systematic review of minor phytocannabinoids with promising neuroprotective potential. Br. J. Pharmacol..

[5] Pennypacker S.D., Romero-Sandoval E.A. (2020). CBD and THC: Do They Complement Each Other Like Yin and Yang?. Pharmacotherapy.

[6] Voicu V., Brehar F.M., Toader C., Covache-Busuioc R.A., Corlatescu A.D., Bordeianu A., Costin H.P., Bratu B.G., Glavan L.A., Ciurea A.V. (2023). Cannabinoids in Medicine: A Multifaceted Exploration of Types, Therapeutic Applications, and Emerging Opportunities in Neurodegenerative Diseases and Cancer Therapy. Biomolecules.

[7] Mori M.A., Meyer E., Soares L.M., Milani H., Guimaraes F.S., de Oliveira R.M.W. (2017). Cannabidiol reduces neuroinflammation and promotes neuroplasticity and functional recovery after brain ischemia. Prog. Neuro-Psychopharmacol. Biol. Psychiatry.

[8] Pazos M.R., Cinquina V., Gomez A., Layunta R., Santos M., Fernandez-Ruiz J., Martinez-Orgado J. (2012). Cannabidiol administration after hypoxia-ischemia to newborn rats reduces long-term brain injury and restores neurobehavioral function. Neuropharmacology.

[9] Valdeolivas S., Navarrete C., Cantarero I., Bellido M.L., Munoz E., Sagredo O. (2015). Neuroprotective properties of cannabigerol in Huntington’s disease: Studies in R6/2 mice and 3-nitropropionate-lesioned mice. Neurother. J. Am. Soc. Exp. Neurother..

[10] Gugliandolo A., Silvestro S., Chiricosta L., Pollastro F., Bramanti P., Mazzon E. (2020). The Transcriptomic Analysis of NSC-34 Motor Neuron-Like Cells Reveals That Cannabigerol Influences Synaptic Pathways: A Comparative Study with Cannabidiol. Life.

[11] Cashman N.R., Durham H.D., Blusztajan J.K., Oda K., Tabira T., Shaw I.T., Dahrouge S., Antel J.P. (1992). Neuroblastoma X Spinal-Cord (Nsc) Hybrid Cell-Lines Resemble Developing Motor Neurons. Dev. Dyn..

[12] Caprioglio D., Amin H.I.M., Taglialatela-Scafati O., Munoz E., Appendino G. (2022). Minor Phytocannabinoids: A Misleading Name but a Promising Opportunity for Biomedical Research. Biomolecules.

[13] Anchesi I., Schepici G., Chiricosta L., Gugliandolo A., Salamone S., Caprioglio D., Pollastro F., Mazzon E. (2023). Delta(8)-THC Induces Up-Regulation of Glutamatergic Pathway Genes in Differentiated SH-SY5Y: A Transcriptomic Study. Int. J. Mol. Sci..

[14] Valeri A., Chiricosta L., Gugliandolo A., Pollastro F., Salamone S., Zingale V.D., Silvestro S., Mazzon E. (2022). Cannabinerol and NSC-34 Transcriptomic Analysis: Is the Dose Who Makes Neuronal Differentiation?. Int. J. Mol. Sci..

[15] Landeira B.S., Santana T., Araujo J.A.M., Tabet E.I., Tannous B.A., Schroeder T., Costa M.R. (2018). Activity-Independent Effects of CREB on Neuronal Survival and Differentiation during Mouse Cerebral Cortex Development. Cereb. Cortex.

[16] Alberini C.M. (2009). Transcription factors in long-term memory and synaptic plasticity. Physiol. Rev..

[17] Durante C., Anceschi L., Brighenti V., Caroli C., Afezolli C., Marchetti A., Cocchi M., Salamone S., Pollastro F., Pellati F. (2022). Application of experimental design in HPLC method optimisation for the simultaneous determination of multiple bioactive cannabinoids. J. Pharm. Biomed. Anal..

[18] Taura F., Morimoto S., Shoyama Y. (1995). Cannabinerolic Acid, a Cannabinoid from Cannabis-Sativa. Phytochemistry.

[19] Hanus L.O., Levy R., De La Vega D., Katz L., Roman M., Tomícek P. (2016). The main cannabinoids content in hashish samples seized in Israel and Czech Republic. Isr. J. Plant Sci..

[20] Bolger A.M., Lohse M., Usadel B. (2014). Trimmomatic: A flexible trimmer for Illumina sequence data. Bioinformatics.

[21] Dobin A., Davis C.A., Schlesinger F., Drenkow J., Zaleski C., Jha S., Batut P., Chaisson M., Gingeras T.R. (2013). STAR: Ultrafast universal RNA-seq aligner. Bioinformatics.

[22] Anders S., Pyl P.T., Huber W. (2015). HTSeq—A Python framework to work with high-throughput sequencing data. Bioinformatics.

[23] Love M.I., Huber W., Anders S. (2014). Moderated estimation of fold change and dispersion for RNA-seq data with DESeq2. Genome Biol..

[24] Gillespie M., Jassal B., Stephan R., Milacic M., Rothfels K., Senff-Ribeiro A., Griss J., Sevilla C., Matthews L., Gong C. (2022). The reactome pathway knowledgebase 2022. Nucleic Acids Res..

[25] Mi H., Ebert D., Muruganujan A., Mills C., Albou L.P., Mushayamaha T., Thomas P.D. (2021). PANTHER version 16: A revised family classification, tree-based classification tool, enhancer regions and extensive API. Nucleic Acids Res..

[26] Koopmans F., van Nierop P., Andres-Alonso M., Byrnes A., Cijsouw T., Coba M.P., Cornelisse L.N., Farrell R.J., Goldschmidt H.L., Howrigan D.P. (2019). SynGO: An Evidence-Based, Expert-Curated Knowledge Base for the Synapse. Neuron.

[27] Cheever T.R., Ervasti J.M. (2013). Actin isoforms in neuronal development and function. Int. Rev. Cell Mol. Biol..

[28] Batool S., Raza H., Zaidi J., Riaz S., Hasan S., Syed N.I. (2019). Synapse formation: From cellular and molecular mechanisms to neurodevelopmental and neurodegenerative disorders. J. Neurophysiol..

[29] Gugliandolo A., Pollastro F., Grassi G., Bramanti P., Mazzon E. (2018). In Vitro Model of Neuroinflammation: Efficacy of Cannabigerol, a Non-Psychoactive Cannabinoid. Int. J. Mol. Sci..

[30] Mammana S., Cavalli E., Gugliandolo A., Silvestro S., Pollastro F., Bramanti P., Mazzon E. (2019). Could the Combination of Two Non-Psychotropic Cannabinoids Counteract Neuroinflammation? Effectiveness of Cannabidiol Associated with Cannabigerol. Medicina.

[31] Valeri A., Chiricosta L., Gugliandolo A., Pollastro F., Mazzon E. (2022). Will Cannabigerol Trigger Neuroregeneration after a Spinal Cord Injury? An In Vitro Answer from NSC-34 Scratch-Injured Cells Transcriptome. Pharmaceuticals.

[32] Qaisar R. (2023). Targeting neuromuscular junction to treat neuromuscular disorders. Life Sci..

[33] Hill M. (2003). The neuromuscular junction disorders. J. Neurol. Neurosurg. Psychiatry.

[34] Gugliandolo A., Blando S., Salamone S., Caprioglio D., Pollastro F., Mazzon E., Chiricosta L. (2023). Delta(8)-THC Protects against Amyloid Beta Toxicity Modulating ER Stress In Vitro: A Transcriptomic Analysis. Int. J. Mol. Sci..

[35] Kim J., Choi P., Park Y.T., Kim T., Ham J., Kim J.C. (2023). The Cannabinoids, CBDA and THCA, Rescue Memory Deficits and Reduce Amyloid-Beta and Tau Pathology in an Alzheimer’s Disease-like Mouse Model. Int. J. Mol. Sci..

[36] Yang S., Du Y., Zhao X., Tang Q., Su W., Hu Y., Yu P. (2022). Cannabidiol Enhances Microglial Beta-Amyloid Peptide Phagocytosis and Clearance via Vanilloid Family Type 2 Channel Activation. Int. J. Mol. Sci..

[37] Hao F., Feng Y. (2021). Cannabidiol (CBD) enhanced the hippocampal immune response and autophagy of APP/PS1 Alzheimer’s mice uncovered by RNA-seq. Life Sci..

[38] Kanchi P.K., Dasmahapatra A.K. (2021). Destabilization of the Alzheimer’s amyloid-beta protofibrils by THC: A molecular dynamics simulation study. J. Mol. Graph. Model..

[39] Paskus J.D., Herring B.E., Roche K.W. (2020). Kalirin and Trio: RhoGEFs in Synaptic Transmission, Plasticity, and Complex Brain Disorders. Trends Neurosci..

[40] Terry-Lorenzo R.T., Torres V.I., Wagh D., Galaz J., Swanson S.K., Florens L., Washburn M.P., Waites C.L., Gundelfinger E.D., Reimer R.J. (2016). Trio, a Rho Family GEF, Interacts with the Presynaptic Active Zone Proteins Piccolo and Bassoon. PLoS ONE.

[41] Montenegro-Venegas C., Guhathakurta D., Pina-Fernandez E., Andres-Alonso M., Plattner F., Gundelfinger E.D., Fejtova A. (2022). Bassoon controls synaptic vesicle release via regulation of presynaptic phosphorylation and cAMP. EMBO Rep..

[42] Waites C.L., Leal-Ortiz S.A., Okerlund N., Dalke H., Fejtova A., Altrock W.D., Gundelfinger E.D., Garner C.C. (2013). Bassoon and Piccolo maintain synapse integrity by regulating protein ubiquitination and degradation. EMBO J..

[43] Hodges J.L., Vilchez S.M., Asmussen H., Whitmore L.A., Horwitz A.R. (2014). alpha-Actinin-2 mediates spine morphology and assembly of the post-synaptic density in hippocampal neurons. PLoS ONE.

[44] Angliker N., Ruegg M.A. (2013). In vivo evidence for mTORC2-mediated actin cytoskeleton rearrangement in neurons. Bioarchitecture.

[45] Mironova Y.A., Giger R.J. (2013). Where no synapses go: Gatekeepers of circuit remodeling and synaptic strength. Trends Neurosci..

[46] Petrinovic M.M., Hourez R., Aloy E.M., Dewarrat G., Gall D., Weinmann O., Gaudias J., Bachmann L.C., Schiffmann S.N., Vogt K.E. (2013). Neuronal Nogo-A negatively regulates dendritic morphology and synaptic transmission in the cerebellum. Proc. Natl. Acad. Sci. USA.

[47] Yao J., Ong S.E., Bajjalieh S. (2014). Huntingtin is associated with cytomatrix proteins at the presynaptic terminal. Mol. Cell. Neurosci..

[48] Barron J.C., Hurley E.P., Parsons M.P. (2021). Huntingtin and the Synapse. Front. Cell. Neurosci..

[49] Kim K., Yang J., Zhong X.P., Kim M.H., Kim Y.S., Lee H.W., Han S., Choi J., Han K., Seo J. (2009). Synaptic removal of diacylglycerol by DGKzeta and PSD-95 regulates dendritic spine maintenance. EMBO J..

[50] Pannaccione A., Piccialli I., Secondo A., Ciccone R., Molinaro P., Boscia F., Annunziato L. (2020). The Na(+)/Ca(2+)exchanger in Alzheimer’s disease. Cell Calcium.

[51] Liao X., Li Y. (2020). Genetic associations between voltage-gated calcium channels and autism spectrum disorder: A systematic review. Mol. Brain.

[52] Andrade A., Brennecke A., Mallat S., Brown J., Gomez-Rivadeneira J., Czepiel N., Londrigan L. (2019). Genetic Associations between Voltage-Gated Calcium Channels and Psychiatric Disorders. Int. J. Mol. Sci..

[53] Paoletti P., Bellone C., Zhou Q. (2013). NMDA receptor subunit diversity: Impact on receptor properties, synaptic plasticity and disease. Nat. Rev. Neurosci..

[54] Bunton-Stasyshyn R.K.A., Wagnon J.L., Wengert E.R., Barker B.S., Faulkner A., Wagley P.K., Bhatia K., Jones J.M., Maniaci M.R., Parent J.M. (2019). Prominent role of forebrain excitatory neurons in SCN8A encephalopathy. Brain.

[55] Nascimento F.A., Andrade D.M. (2016). Myoclonus epilepsy and ataxia due to potassium channel mutation (MEAK) is caused by heterozygous KCNC1 mutations. Epileptic Disord. Int. Epilepsy J. Videotape.

[56] Jeremic D., Sanchez-Rodriguez I., Jimenez-Diaz L., Navarro-Lopez J.D. (2021). Therapeutic potential of targeting G protein-gated inwardly rectifying potassium (GIRK) channels in the central nervous system. Pharmacol. Ther..

[57] Ames J.B. (2021). L-Type Ca(2+) Channel Regulation by Calmodulin and CaBP1. Biomolecules.

[58] Liang Z., Zhan Y., Shen Y., Wong C.C., Yates J.R., Plattner F., Lai K.O., Ip N.Y. (2016). The pseudokinase CaMKv is required for the activity-dependent maintenance of dendritic spines. Nat. Commun..

[59] Oku Y., Huganir R.L. (2013). AGAP3 and Arf6 regulate trafficking of AMPA receptors and synaptic plasticity. J. Neurosci. Off. J. Soc. Neurosci..

[60] Llamosas N., Arora V., Vij R., Kilinc M., Bijoch L., Rojas C., Reich A., Sridharan B., Willems E., Piper D.R. (2020). SYNGAP1 Controls the Maturation of Dendrites, Synaptic Function, and Network Activity in Developing Human Neurons. J. Neurosci. Off. J. Soc. Neurosci..

[61] Yang R., Feng X., Arias-Cavieres A., Mitchell R.M., Polo A., Hu K., Zhong R., Qi C., Zhang R.S., Westneat N. (2023). Upregulation of SYNGAP1 expression in mice and human neurons by redirecting alternative splicing. Neuron.

[62] Takahashi K., Yamanaka S. (2006). Induction of pluripotent stem cells from mouse embryonic and adult fibroblast cultures by defined factors. Cell.

[63] Borgmann-Winter K.E., Wang H.Y., Ray R., Willis B.R., Moberg P.J., Rawson N.E., Gur R.E., Turetsky B.I., Hahn C.G. (2016). Altered G Protein Coupling in Olfactory Neuroepithelial Cells From Patients With Schizophrenia. Schizophr. Bull..

[64] Bellon A., Wegener A., Lescallette A.R., Valente M., Yang S.K., Gardette R., Matricon J., Mouaffak F., Watts P., Vimeux L. (2018). Transdifferentiation of Human Circulating Monocytes Into Neuronal-Like Cells in 20 Days and Without Reprograming. Front. Mol. Neurosci..

