# Supplementary figures and images for: Cannabinerol (CBNR) Influences Synaptic Genes Associated with Cytoskeleton and Ion Channels in NSC-34 Cell Line: A Transcriptomic Study
Source: Biomedicines. 2024 Jan 15;12(1):189. doi: 10.3390/biomedicines12010189 (PMC10813620; doi:10.3390/biomedicines12010189)

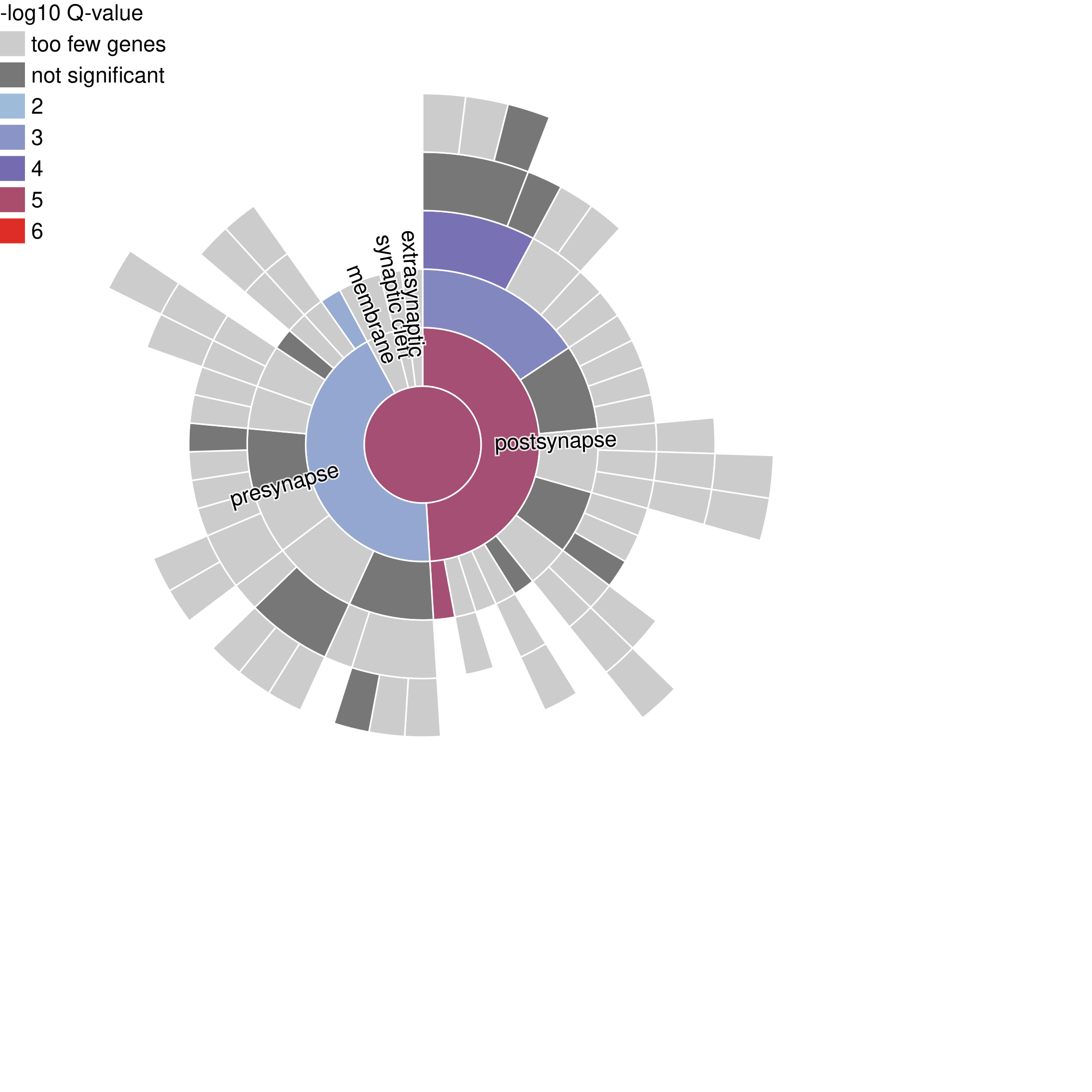

Supplement: Supplementary file 1 [file biomedicines-12-00189-s001.zip › Figure S1.tiff]

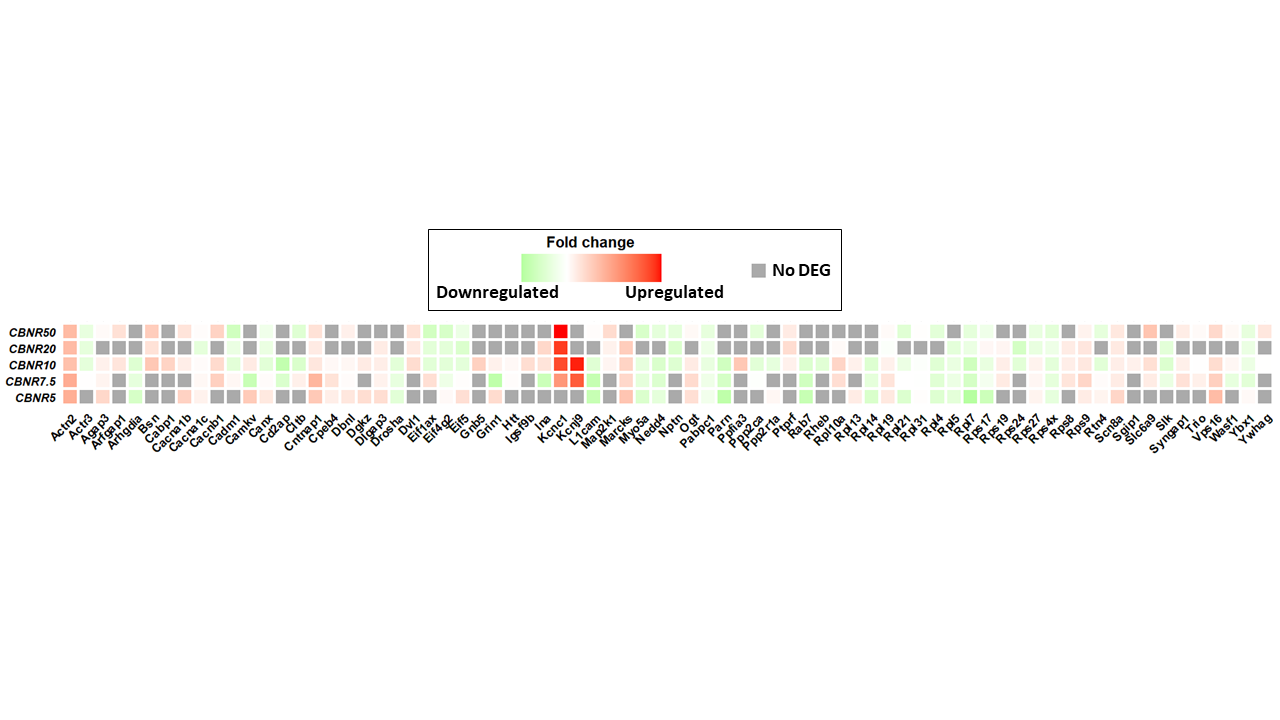

Supplement: Supplementary file 1 [file biomedicines-12-00189-s001.zip › Figure S2.tif]
